# ConTra Preference Language: Privacy Preference Unification via Privacy Interfaces

**DOI:** 10.3390/s22145428

**Published:** 2022-07-20

**Authors:** Stefan Becher, Armin Gerl

**Affiliations:** Chair for Distributed Information Systems, Faculty of Computer Science and Mathematics, University of Passau, 94032 Passau, Germany

**Keywords:** privacy, preference language, legal factors, GDPR, usability

## Abstract

After the enactment of the GDPR in 2018, many companies were forced to rethink their privacy management in order to comply with the new legal framework. These changes mostly affect the *Controller* to achieve GDPR-compliant privacy policies and management.However, measures to give users a better understanding of privacy, which is essential to generate legitimate interest in the *Controller*, are often skipped. We recommend addressing this issue by the usage of privacy preference languages, whereas users define rules regarding their preferences for privacy handling. In the literature, preference languages only work with their corresponding privacy language, which limits their applicability. In this paper, we propose the ConTra preference language, which we envision to support users during privacy policy negotiation while meeting current technical and legal requirements. Therefore, ConTra preferences are defined showing its expressiveness, extensibility, and applicability in resource-limited IoT scenarios. In addition, we introduce a generic approach which provides privacy language compatibility for unified preference matching.

## 1. Introduction

While the enactment of the GDPR in 2018 within the European Union affected the privacy management of many service providers, the intended effect of the GDPR did not propagate to the users. Therefore, privacy policies remain to be an overwhelming barrier to understand [1]. Due to the manifold purposes and the legal relevance of privacy policies, many consist of several pages of content written in a highly technical style by domain experts, which is too complex for users to understand and in the end may not even sufficiently answer the question “Is my privacy protected?”. This has been confirmed by McDonald and Cranor [2], who estimated that reading a privacy policy takes about ten minutes on average. Linden et al. [1] showed that after the enactment of the GDPR, privacy policies have grown in size. Further challenges emerge from different technical environments, such as IoT, where connections between devices can be dynamically created or changed and edge devices often do not have appropriate capabilities to display information or gather explicit consent to policy actions by a user input [3].

Although privacy policies are legally regulated, only a few attempts have been made to standardize them in a common format in order to overcome this complexity and to increase interoperability. One of these attempts are privacy languages, e.g., P3P [4], PPL [5], LPL [6], and SPECIAL [7]. While these works demonstrate that the formalization of privacy policies is viable, they are not used in practice. Even P3P, as the most used privacy language, has not been supported by user agents since 2018 [4] due to the lack of legal incentives for service providers.

Privacy policies can be personalized by the user regarding their preferences before agreeing to it, which in the following is denoted as policy negotiation. This is usually implemented by optional purposes, e.g., access to the GPS data of the device, which are not mandatory for a service but may provide benefits, e.g., a recommendation for a great restaurant in the area. The application of a preference language, such as APPEL [8], XPref [9], or YaPPL [3], could support this step. The latter would require users to invest time into a preference setup process which then can be matched against privacy policies and help users at negotiation. Research has shown that this is a critical step because, due to the *Privacy Paradox* [10], users are concerned about privacy but are not willing to spend much time on its management. To tackle this problem, measures need to be taken to make information about data protection more accessible.

With our work, we focus on the following research question:

*“What are the requirements for a post-GDPR privacy preference language that enables the negotiation with privacy policies, and do state-of-the-art preference languages fulfill these requirements?”*

To answer this research question, we introduce a use case, define requirements for a post-GDPR preference language, and survey related work based on it in Section 2. Existing preference languages are designed to complement exactly one privacy language. As privacy languages differ in terms of their syntax and semantics, many privacy policies would not be affected by a specific preference language setup. In addition, no standardized vocabulary for privacy expressions, which would help to create unified policies, is shared between different implementations. Currently, no preference language is able to deal with such a situation. This leads to the main contribution of this work, which is the definition of ConTra (a preference language for **Con**sent and **Tra**nsparency) in Section 3. Therefore, the aforementioned gaps, i.e., missing privacy language compatibility and standardization, are addressed by the introduction of unified privacy interfaces in Section 4. ConTra is designed to fulfill legal requirements defined by the GDPR; it supports mobile technologies by defining preferences for context information, such as location and time, works in resource-limited IoT environments and also considers the human-readability of preference rules. With the broad amount of applications ConTra is able to cover, we hope to raise the awareness of users about the importance of privacy management and motivate further research in the field of preference languages. Finally, in Section 5, we discuss the results of the paper and answer the research question. Section 6 concludes the work.

## 2. Background

When users are presented with a privacy policy, there are basically two distinct layers of options to interact with the content of the policy. First, a plain-text privacy policy is presented to the user, which either might be completely accepted or rejected. Based on this selection, a service might either be usable or not.

Second, a privacy policy contains several purposes, whereas each purpose defines the processing of personal data by a *Controller* for a specific use. A purpose can be mandatory, e.g., a user must share information about his address for shipment when purchasing products in a web store, or optional, e.g., a user might also share his GPS data to obtain recommendations of nearby physical stores. To accept a privacy policy, all mandatory purposes must be agreed to, whereas optional purposes may be agreed to based on the user’s preferences. This is how privacy policies are personalized. In this work, we focus on optional purposes and their negotiation.

**Figure 1:**
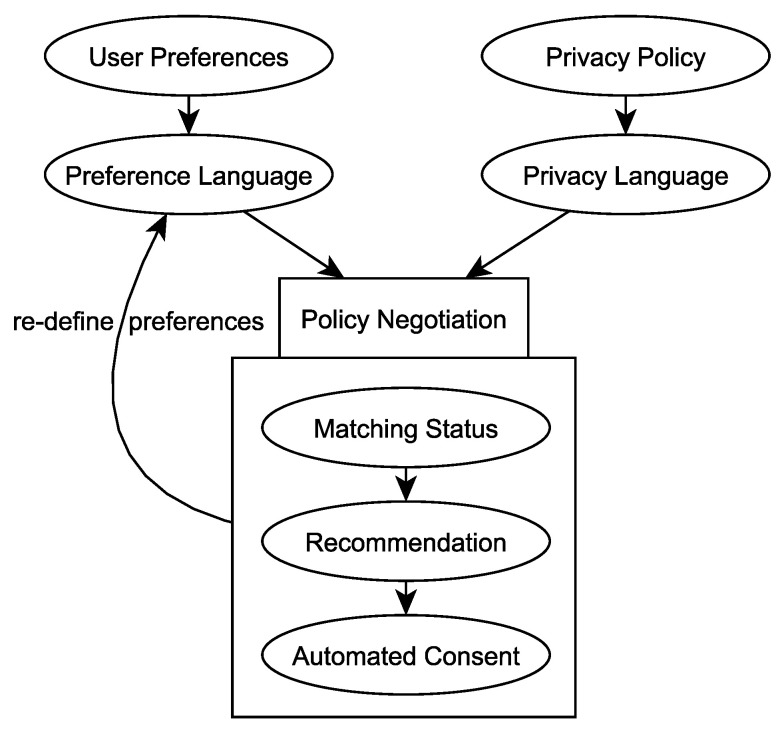
User preferences, expressed in preference languages, are matched against the privacy policies of *Controllers*, which are expressed in privacy languages. Matching status, consent recommendation or automated consent are used to ease policy negotiation.

As this is a rather complex process, both privacy languages and preference languages aim to support users at managing their consent during privacy policy negotiation (see Figure 1). Therefore, preference languages are technical representations of user preferences, and privacy languages technically express privacy policies. These representations are matched against each other, whereas policy negotiation will be eased by giving the user information about the matching status, recommending certain consent decisions or even automatically giving consent. Additionally, the preferences can be extended or altered, based on the matching results, to achieve a more precise matching processes in the future.

### 2.1. Negotiation Automation Levels

It must be considered that the more support that is provided by technical measures, the more the privacy policy negotiation will transform into a (semi-)automated process. Thus, we envision the following five levels of policy negotiation automation:

- *Level 1*—Manual fine-grained consent: This is the very basic case where only the privacy policy is given. Users need to inform themselves about the policy content and can personalize the policy by either accepting or rejecting optional content manually, i.e., give consent. Finally, the user can agree to the policy. In this case, no tools support the process.
- *Level 2*—Manual consent profile selection: As on *level 1,* the privacy policy is given and users have to inform themselves about its content. Policy negotiation support is offered by providing predefined privacy profiles, e.g., predefined by the *Controller*, which users can select and possibly personalize. Based on the selected profile, users agree to the policy.
- *Level 3*—Preference-based consent negotiation: *Level 3* introduces user preferences which are defined in an initial setup process. During policy negotiation, the predefined preferences are compared against the policy, which results in either a match or mismatch. If a mismatch happens, users usually receive hints about what parts of the policy did not match the preferences. Based on this information, users can personalize the privacy policy and make their decision if they agree to it.
- *Level 4*—Preference-based consent recommendation: Similar to *level 3*, user preferences are defined in a setup process beforehand. Privacy policies are then analyzed during policy negotiation and compared against the predefined preferences. As a result, recommendations are given to users regarding how to personalize the policy. Users can then decide whether to apply the suggested options or not and agree to the policy by themselves.
- *Level 5*—Preference-based automated consent: As with *level 4*, predefined preferences are compared to the privacy policy during negotiation. Then, the policy is automatically personalized based on the comparison, and consent is given automatically. In this case, there is no user interaction at policy negotiation at all.

This taxonomy shows how policy negotiation can vary from a manual process to full automation by the support of proper technologies.

### 2.2. Preference Language Requirements

In this work, we focus on *level 3* and *level 4* of the negotiation automation levels. Based on the levels, we now define requirements that a modern preference language should fulfill. For the most part, the requirements are based on a recent survey regarding preference language modeling and adoption conducted by Kapitsaki et al. [11]. Additionally, we included legal requirements (namely the consideration of the GDPR) as well as the research gap, which we identified for privacy language compatibility.

**Privacy language compatibility.** To the best of our knowledge, all existing approaches for preference languages share the same drawback: that they can negotiate only the privacy language for which they were designed. We envision a novel preference language to cover a huge part of the privacy sector. To realize this vision, preferences should be compatible with many privacy languages, as we see the limited applicability of existing preferences as a main problem that causes the minor usage. As each privacy language is represented in a different syntax and offers specific functionalities, standardization is needed, which is often missing in both privacy policies and preferences.

**Legal situation.** To deal with the current legal framework defined by the GDPR [12], privacy preferences need to support explicit consent by design [12] (Art. 7). Consent or dissent is modeled by preferences, and information or recommendations about policy matching will be given to the user. This represents *level 3* and *level 4* of the negotiation automation levels. The consent recommendations can then be applied or customized by the user to achieve informed lawfulness of processing [12] (Art. 6 No. 1). No consent is automatically given at policy negotiation. In addition, policy actions should be presented to users in a transparent way. There are technical solutions to check if real applications comply with this requirement; e.g., PADRES [13] is a tool that provides an extensible questionnaire regarding GDPR compliance combined with several tools for security analysis, or privacyTracker [14], which supports data traceability.

**Transparency.** While transparency is also part of legal requirements [12] (Art. 12–14), we consider it to be essential to state it as an independent requirement. Users should be aware of policy actions, and no actions should be hidden from the user behind huge text blocks written in expert knowledge. To inform users about their data subject rights [12] (Art. 15–23), information should be provided in easily understandable representations. Therefore, a preference language must provide summaries of relevant policy statements, especially those that contradict with the user preferences. In addition, privacy preference rules need to be human-readable, so that the statement of the preference is clear to the user.

**Human- and machine-readability.** The setup of preferences needs to be designed so it can be done easily and quickly by the user in order to address a huge audience with different technical and legal knowledge. This means that human-readability needs to be enabled and facilitated by the syntax and semantics of the preference language. In addition, machine-readability must be given for efficient processing of the preference rules at negotiating privacy languages, which should also work in resource-limited environments, such as IoT scenarios. This might conflict with the required human-readability. Therefore, a representation of preferences should be used that fulfills both of these requirements to a certain degree.

**Expressiveness.** If a user is not able to express his visions in one or more preference rules, then the preference language will ultimately fail. To gain high expressiveness, preferences should be built upon a generic basis and need to be able to support standardized privacy vocabularies. It needs to be considered that too high expressiveness might overwhelm the user, while too few options might cause the user to not be able to define the desired rules. Therefore, predefined placeholders assist at formulating general rules, which can quickly be defined, but also very specific rules can be defined, which utilize the full extent of the preference language. As a result, preferences are not restricted to a user type or a specific domain, such as IoT, e-commerce or e-health, but they can be applied on any scenario.

**Context information.** To cover mobile applications, which may claim access to data unique to mobile devices, such as GPS data, a modern preference language should be capable of expressing context information in preferences. In addition to location information, also, time constraints are an interesting application of this feature. Therefore, data usage by *Controllers* can be limited to certain areas or time-spans. One could also think of setting constraints for specific networks, e.g., a company intranet, to limit data access to certain sub-nets.

**Extensibility.** Preference rules should be applicable to multiple domains. The syntax of the preference rules must by defined in a generic format, so that it allows for extensions, which result from requirements of new application fields. For example, rules could be extended by options for accountability. Therefore, the evaluation of the rules must be able to adapt to newly introduced extensions. In addition, standardized, semantic representations of preference elements help for maintaining extensibility.

### 2.3. Related Work

In the following, related work will be discussed based on the policy negotiation automation levels and in case of preference languages also regarding the above-mentioned requirements. The results of the latter are summarized in chronological order by the publication date of the preference language in Table 1.

In current state-of-the-art of policy negotiation, users commonly face *level 1* of policy negotiation automation almost all the time, as most of the service providers do not offer additional help and tools supporting higher levels of automation are not given for real applications to the best of our knowledge. P3P [4] is a well-known privacy language which has been also used in practice by several service providers. Its main usage is to create machine-readable privacy policies and to inform users about the policy content. E-P3P [15] is an extension to P3P to represent and enforce claims of P3P policies internally in enterprise environments. Since the development of P3P, many privacy languages came up, whereas each of them is designed for a specific application scenario. Just to name a few, P2U [16] supports secondary user data sharing across different platforms, EPAL [17] represents internal policies of enterprises, and LPL [6] provides the “LPL Policy Viewer” [18], which creates a policy summary based on purposes and allows for detailed personalization. In general, most of the established privacy languages work on this level. The three common choices a user has at this level are to fully accept, fully reject, or personalize the optional purposes of a privacy policy. Even though these languages try to make policies easier to understand and offer various advantages for background processing, the negotiation step is still a process the user has to overcome without further help.

While *level 2* of the negotiation automation level still represents only the usage of privacy languages, more tool support is provided compared to *level 1*. Therefore, users have access to so-called privacy profiles, which represent predefined choices for consent options based on various levels. Users can select a profile level of their liking and customize it instead of manually having to select all choices by their own. Choices are usually grouped together by topics, which makes it easier for the user to include or exclude certain functionality, e.g., advertisement or GPS tracking. This allows for a faster and better-informed policy negotiation. An example for a *level 2* application is the “Consent and Control UI Prototype 3” as part of the SPECIAL project [7]. Via a slider, more usage purpose groups (each representing a privacy profile level) are selected to be consented to, from required purposes to optional. Within the groups, it is possible to manually withdraw consent for each of the pre-selected choices. Although these solutions provide faster policy negotiation, it remains a manual process, whereas the user needs to evaluate for each new privacy policy if it meets his preferences.

This problem, i.e., manual preference comparison, is tackled by *level 3* of the negotiation automation levels, which represents most of the research conducted for preference languages. Often, this level is supported by UI tools, but there are also approaches without. As the main focus of this paper is about preference languages, the most well-known of their kind, namely APPEL [8], XPref [9], SemPref [19], SecPAL4P [20], PPL [5], CPL [21], and YaPPL [3], will be discussed in the following based on the requirements. The results are summarized in Table 1. An early example for creating transparency is Privacy Bird [22], which summarizes P3P [4] policies and highlights mismatches based on APPEL preferences. As many later approaches build upon APPEL and P3P, e.g., XPref and SemPref, or are inspired by it, the transparency of policy actions is an important criterion for almost all preference languages. Only CPL and YaPPL are missing this point, because their research did not include representations of privacy policies. Research uncovered weaknesses of P3P and APPEL regarding expressiveness and extensibility [9]. XPref and SemPref also lack these criteria, because they use APPEL as a basis. Newer languages tried to fix this problem and set a strong focus on both expressiveness and extensibility. This trend is clearly visualized by the overview table. However, one needs to be careful, because too high expressiveness often results in too complex rule formulation, as seen with generic SecPAL4P [20] preferences, while simple rules are often too limited, as shown for APPEL [8]. Therefore, a preference language must fulfill both sides, i.e., simplicity of rule formalization and good expressiveness. An example for extensibility is PPL, which has been extended to A-PPL [23] by adding accountability obligations to the preference rules. As all the preference languages are machine-readable, we did not include this column in the overview. However, it is worth mentioning that some of them are designed to specifically work in resource-limited environments, e.g., YaPPL preferences for IoT edge devices, while others aim to support common browsing on websites. For human-readability, there is no clear trend. Most preference languages are based on XML representations, which are hard to understand for users. SemPref has an additional representation specifically designed for human-readability, and YaPPL preferences are based on the JSON format, which is easy to understand by users. In our opinion, a modern preference language should provide both efficient processing and human-readability, because IoT and smart sensor devices are widely used, and preferences should be understandable by users even without graphical interfaces. It also shows that more recent preference languages set a stronger focus on legal requirements and expressing context information. This can be explained by strengthened legal restrictions, such as the enactment of the GDPR, and the rise of mobile devices and applications. YaPPL models enable explicit consent and dissent in preferences as requested by the GDPR, while CPL is specifically designed to introduce context information, namely time and location, in preferences. PPL fulfills both criteria to a certain degree by authorization techniques and introducing obligations into preference rules. While each of these preference languages serves a certain purpose, all of them were designed to complement a specific privacy language. This limits their applicability, as privacy policies can be defined by different privacy languages, and therefore, preferences cannot negotiate with all of them. Thus, the requirement of privacy language compatibility is not met in the current state-of-the-art. We define this absence of compatibility as the first research gap regarding preference languages.

Automation *level 4* represents recommendations, whereas consent choices at policy negotiation will be pre-selected based on the preferences of the user. Even though this could be featured by a preference language, to the best of our knowledge, none of the existing preference languages can produce automated recommendations for consent negotiation. We identify this as the second research gap regarding preference languages for policy negotiation. Nevertheless, there are some independent recommendation systems that work on *level 4*; e.g., Sanchez et al. [24] propose an approach to predict user choices for fitness app permissions by unsupervised machine learning, but the solution is limited to this use case and needs to be extended to many/all others. This is questionable, because data for machine learning may not be available in every use case or one-time life event, e.g., severe illness, etc.

Automated consent based on user preferences without interaction of the user is provided by *level 5* of the negotiation automation levels. It is questionable if this level is reachable under the current legal framework of the GDPR. It is not clearly defined if users have to give consent explicitly by their own action or if this action can be made by a third party or proxy tool based on the user’s preference decisions [12] (GDPR Art. 6 No. 1 “Lawfulness of processing”). This leaves room for discussion and interpretation, which has to be completed by the legal community first. Thus, we state the open question: is consent generated by automated policy negotiation legally compliant with the GDPR framework in Europe?

To sum up, we identified missing privacy language compatibility and missing consent recommendation for privacy policy negotiation as two research gaps regarding privacy preference languages.

## 3. ConTra Preference Language

In order to overcome the aforementioned research gaps regarding preference languages, we propose ConTra (a preference language for **Con**sent and **Tra**nsparency). ConTra is a privacy preference language which is designed to meet the requirements defined for a modern preference language. The following sections will introduce the concept of ConTra in detail, whereas the preference setup is described first, which is followed by the functionality of policy negotiation and the usage of Deontic Logic within this process. Next, the structure and elements of ConTra are modeled.

### 3.1. Concept

With ConTra, we propose a preference language which is easy to use and covers a broad amount of applications by providing privacy language compatibility in a generic way. In addition, it has a close focus to the legal side of privacy, as its name already indicates. It should assist users in making their consent decision to the content of privacy policies while informing them about privacy policy actions in a transparent way.

#### 3.1.1. Preference Setup

During an initial setup process, users are able to formulate preferences in the ConTra language, which are machine-readable representations of the user’s preferences regarding privacy policies. This feature can be found in APPEL [8], PPL [5], and YaPPL [3], too. The definition of preferences is a critical step in the usage process of privacy preferences. Research has shown that users are not willing to invest time into an extensive setup process for privacy [25]. Therefore, we aim for a very basic, short initial definition of preferences. Predefined preference profiles, which can be customized, assist at this step. The usefulness of these profiles will be evaluated in future work. Users will have the opportunity to create, alter and extend rules whilst negotiating a privacy policy. This way, we try to stretch the setup process over several sessions and aim for a better acceptance, even though the evaluation of this feature is part of future work, too. Preference rules will be defined in JSON format for both human- and machine-readability. For the first prototype of ConTra, the definition of preferences is manually conducted. In the future, the development of privacy-enhancing tools will assist at this step.

#### 3.1.2. Policy Negotiation

While users are negotiating with a privacy policy, the predefined preferences will simplify this process. The preference rules, which a user generated in advance, and the policy are matched against each other. Then, information about the matching status is given to the user. It is worth noting that an interaction between preferences and a policy is only possible if both are available in a machine-readable way, i.e., a plain text privacy policy could be analyzed by text mining approaches and transformed in a privacy language representation, or a simpler privacy language is used. We assume the usage of privacy languages for simplicity for this paper to make the whole concept work. ConTra is able to interact with multiple privacy languages to gain a huge coverage of websites that support ConTra preferences, while most existing preference languages are specifically designed to work with only one privacy language, thus limiting their applicability. This is enabled by a unified registry that is based on a standardized privacy vocabulary in which data protection officers can integrate unique privacy language constructs (see Section 4). Information is given to the user after the preference matching is completed. Users will be informed if their preferences are matching the underlying privacy policy on visiting a website that is based on a supported privacy language. A later added browser extension, the *ConTra Negotiation UI*, will visualize the matching status for a very brief overview. This feature is inspired by Privacy Bird [22], which was developed for P3P. Studies of Cranor et al. [22] have shown that by using the tool, users actually tend to inform themselves more about privacy policy actions. As several established or modern privacy languages feature measures or can be supported by tools to visualize the content of privacy policies in transparent and easily understandable ways, one task a preference language should realize is to motivate users to look up this information on their own. For example, Privacy Bird [22] has a function to summarize the content of a P3P privacy policy, and Polisis [26] uses machine learning to analyze plain text privacy policies and to visualize the most important parts of the gained information. Future work will show if one of the existing approaches for policy summary can be integrated into the ConTra framework or if we will develop our own approach.

While users are in the process of negotiating a privacy policy, the *ConTra Consent Recommender UI* shows a detailed matching status of the preferences and policy. At this step, clearly matching purposes are preselected by the tool, and the user only has to check for correctness or purposes without predefined preferences. In addition, users have the possibility to extend the preference rules by missing purposes for future negotiation processes. It should be mentioned that no automated consent will be given. Even if all purposes match the privacy policy, the user still must check the recommendation and actively give consent by himself. As a result, the overall privacy policy negotiation will be accelerated and happen on a more informed basis on the side of the user.

#### 3.1.3. Deontic Logic

For the formalization of ConTra preferences, we decided to adapt parts of Deontic Logic [27]. Therefore, the phrases *permissible* (which we call *permitted*) and *impermissible* (which we call *excluded*) will be utilized to create two distinct collections for the elements of a preference rule. This separation into *permitted* and *excluded* elements comes with several advantages. First, while the rules of most existing preference languages can be only either of an accepting or rejecting nature, such as APPEL [8] and PPL [5], and multiple rules are needed to model both criteria, only a single ConTra rule is needed to model the same functionality. For example, one can define a ConTra preference that allows the usage of GPS data for a specific service provider but forbids marketing. In APPEL, this could only be expressed as an accepting preference for GPS data and a separate rejecting preference for marketing. By defining items in either the *permitted* or the *excluded* collection of a *Rule*-element, explicit consent or dissent, as requested by the GDPR, is modeled. The requirement for considering the legal situation is therefore fulfilled. Finally, the usage of a predefined placeholder with the meaning *all* either accepts or rejects every item of a collections category. For example, a user can define a preference to allow all possible data elements for the purpose “Research” by setting the *permitted* collection of the data element to *all*. This leads to high expressiveness and easily definable preferences. The YaPPL preference language [3] also provides the same separation in *permitted* and *excluded* collections and has shown that it is a valid concept.

### 3.2. Structure and Elements

**Figure 2:**
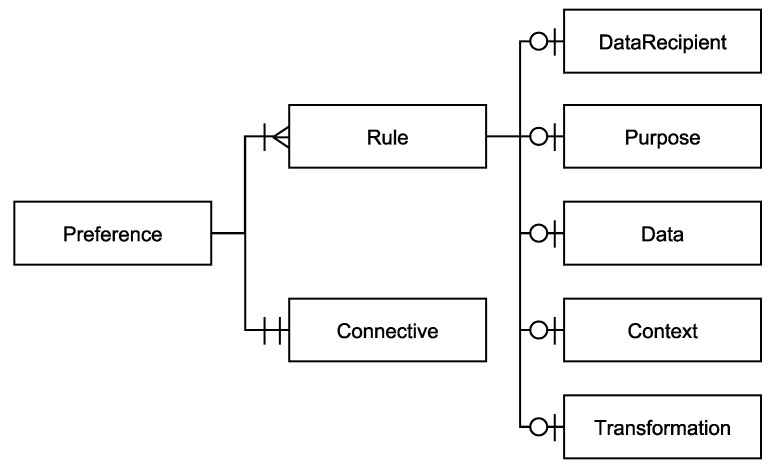
Hierarchical structure of the elements of a ConTra preference. Attributes are omitted from this figure.

In the following, the structure of a ConTra preference is detailed as well as its elements and their most important attributes. ConTra preferences are structured hierarchically based on the JSON format (see Figure 2) to maintain both human- and machine-readability. A ConTra preference with the *Preference*-element as its root can contain several *Rule*-elements that are logically connected with each other by a *Connective*-element. This allows for rule combinations within a single preference, whereas the requirement for high expressiveness is given. The *Rule*-element contains the *DataRecipient-, Purpose-, Data-, Context-, and Transformation*-element. By design, all the elements at the bottom of the hierarchy are optional. This is possible, because all the elements are independent of each other and the evaluation algorithm of ConTra preferences is able to process preferences if the *Rule*-element contains at least one of the bottom hierarchy elements. Therefore, very general rules, which are easy to understand by the user, can be defined, e.g., one that only excludes the usage of location data. In this case, the usage of location data is always forbidden, i.e., for any *DataRecipient*, *Purpose*, etc. In addition, very specific preferences can be defined, which utilize all of the possible elements. By keeping the elements optional and independent of each other, future extensions can easily be integrated, because only the evaluation algorithm needs to be extended by the newly introduced element. Therefore, the requirement for extensibility is also given by the structure of ConTra.

Now, the elements of a ConTra preference are detailed based on the structure of Figure 2, whereas each element is defined by its attributes.

**Preference.** The *Preference*-element as the root capsules the rules of a single ConTra preference. It is defined as *Preference = ([Rule], Connective, version, id, validFrom, validTo, description)*, whereas *[Rule]* is denoted as a list of *Rule*-elements.The elements of *[Rule]* are combined by a logical operation defined in the *Connective*-element. Within a single *Preference*-element, there is no limit for *Rule*-elements, but they are all joined together by the same *Connective*. A *Preference* also contains the attributes *version* for future ConTra version management, *validFrom* and *validTo* for tracking the validity of the preference, *id* for preference management and finally *description* as a human-readable, textual description of the preferences’ meaning.

**Connective.** The *Connective*-element is used within a *Preference* to join multiple *Rule*-elements together. It is defined as *Connective = (operator)*. *Connective* contains a single logical operator, whereas currently *AND* and *OR* are supported. If the desired preference contains three or more *Rule*-elements and would need to utilize both the *AND* and *OR* operator, then it should be split and defined in a second *Preference*-element in order to keep the creation process clear. In addition, a *Connective* is only needed if more than one *Rule*-elements have been defined. Otherwise, it will be ignored. To ensure that no contradictions are created by joining rules together, a validation algorithm will check the validity of created preferences.

**Rule.** The *Rule*-element defines the behavior of a ConTra preference. It contains restrictions and permissions about privacy policy actions that are used to support the negotiation process of users with privacy policies. This element is defined as *Rule = (DataRecipient, Purpose, Data, Context, Transformation)*. The combination of these elements forms a ConTra preference, reflecting a user’s vision of how his personal data should be processed. It is worth noting that none of the components of the *Rule*-element is obligatory. In addition, there is no ordering or logical relationship between the components.

**DataRecipient.** The *DataRecipient*-element defines *Controllers*, in this case a legal entity, which are allowed or rejected to collect and process the personal data of the user. It is defined as *DataRecipient = ([permitted_recipient], [excluded_recipient], thirdPartySharing)*. It utilizes a *permitted_recipient* and an *excluded_recipient* collection based on the already introduced Deontic Logic model. This results in two distinct collections, one containing *Controllers* that are explicitly accepted by the user to be allowed to collect data and one containing *Controllers* that are explicitly forbidden to do so. In addition, the usage of the placeholder *all* either explicitly allows or forbids collecting of data for all possible *Controllers* within one of the two collections. Therefore, explicit consent is modeled for any case. In addition to the two collections, the *DataRecipient*-element also has the *thirdPartySharing*-attribute that lets the user decide if personal data should be allowed to be shared with third party *Controllers*. This attribute is less weighted than the two collections, meaning that for example, a third party *Controller*, which is explicitly permitted to collect personal data, will not lose the permission if *thirdPartySharing* is set to false and vice versa.

**Purpose.** The *Purpose*-element defines usage purposes for which the collection and processing by a *Controller* is either *permitted* or *excluded*. It is defined as *Purpose = ([permitted_purpose], [excluded_purpose])*. This element also contains two distinct collections, namely *permitted_purpose* and *excluded_purpose*, that follow the same principle as with the *DataRecipient*-element. Therefore, a standardized purpose “Research” might be permitted, while “Advertising” might be a rejected purpose for a preference.

**Data.** The *Data*-element defines which data categories, such as age or gender, are allowed to be processed by an otherwise permitted *Controller* and which are not. This element is defined as *Data = ([permitted_data], [excluded_data])*. Again, the Deontic Logic model is used as a basis. Therefore, the *Data*-element contains the collections *permitted_data* and *excluded_data*. For example, a preference could be defined, which allows the usage of the age but forbids the usage of location data for certain purposes.

**Context.** The *Context*-element defines in which environmental context the collection of personal data is allowed or rejected by a permitted *Controller*. It is defined as *Context = (time_period, [location])*. The *Context* can either be set to time or location information. Time constraints define points in time or time periods at which data collection is allowed by *Controllers*. Location constraints define places on earth where data collection is allowed by the *Controller*. Examples are the user’s home country or regions with the same legal framework, such as the European Union. The latter option could be useful when it comes to detecting GDPR-compliant privacy policies. It should be noted that most privacy languages do not (fully) support context information yet. An example is LoPSiL [28], which is a privacy language specifically designed to express location-dependent privacy policies, although it does not support time constraints. Nevertheless, because of the huge mass of mobile devices currently in use and the fact that IoT services are used more with the growing popularity of smart homes, in our opinion, modern privacy languages and preference languages should both feature context information in order to cope with this situation. Therefore, ConTra already supports context information in its preferences, hoping that it inspires further research on its integration into privacy languages.

**Transformation.** The *Transformation*-element defines methods that are used for the de-identification of personal data before they are processed by a permitted *Controller*. It is defined as *Transformation = (transformation_level)*. By taking use of this option, a user will be warned if his data will be analyzed without masking his identity. At the current state of ConTra, this element allows for a single choice for *transformation_level* between *no_method*, *pseudonymization*, *anonymization* and *any_method*. At a later point, we will evaluate if it is also sufficient or even more intuitive to limit the choice to whether means for data de-identification should be taken or not.

### 3.3. Use Cases

The application of a privacy preference language is not limited to a single use case. In fact, there are plenty of suitable scenarios where a preference language can support users or even companies. As a first scope of ConTra, we see the integration into a personal computers browser in order to assist users while doing their daily browsing on a static workplace. For example, let us assume a user uses his browser mainly for online shopping. Therefore, he defines a preference, which allows the usage of the *Data*-elements “Address” and “Financial Data” for any recipient specifically for the purpose “E-Commerce” and explicitly forbids the usage for every other purpose (see Listing 1). If the *Controller* would use one of the allowed *Data*-elements for another purpose than “E-Commerce” or process additional *Data*-elements, then the user will be warned during policy negotiation.

Listing 1 ConTra preference for sharing address and financial data for e-commerce, while forbidding usage for any other purpose. The symbol * represents the placeholder *all*.

1 "preference" : {
2   "connective" : AND,
3   "rule" : {
4     "purpose" : {
5         "permitted" : ["E-Commerce"],
6         "excluded" : [*]
7     },
8     "data" : {
9         "permitted" : ["Address", "Financial Data"],
10         "excluded" : [ ]
11     }
12   }
13 }

At a later point, the user decides that he wants to receive some recommendations for products while shopping but only within the European Union. He extends the original preference by a second rule, which allows the usage of all *Data*-elements for the *Purpose* “Product Recommendation” and sets the *Context* to “European Union”. In addition, he does not want the data used for recommendation to be shared with other *Controllers* and therefore sets *thirdPartySharing* to “false” (see Listing 2). He will be warned again if some behavior of the *Controller* mismatches the newly added rule. Therefore, negotiation with the privacy policies of websites will be eased, and users obtain a more transparent and informed insight into the data-handling practices of the service provider.

Listing 2 ConTra preference of Listing 1 extended by allowing usage of any data for product recommendation within the European Union but not for third party . The symbol * represents the placeholder *all*.

1 "preference" : {
2   "connective" : AND,
3   "rule" : {
4     "purpose" : {
5         "permitted" : ["E-Commerce"],
6         "excluded" : [*]
7     },
8     "data" : {
9         "permitted" : ["Address", "Financial Data"],
10         "excluded" : [ ]
11     }
12   },
13   "rule" : {
14     "dataRecipient" : {
15         "thirdPartySharing" : "false"
16     },
17     "purpose" : {
18         "permitted" : ["Product Recommendation"],
19         "excluded" : [ ]
20     },
21     "data" : {
22         "permitted" : [*],
23         "excluded" : [ ]
24     },
25     "context" : {
26         "region" : "European Union"
27     }
28   }
29 }

The usage in mobile environments will be the second scope of ConTra. In this case, there are also some interesting scenarios where companies can take advantage of preferences. Let us assume an individual who carries a mobile device defines a preference utilizing the *Location-Context* that allows data collection by his employer while being on the company grounds. In this case, the employer can collect the needed information for business processes while the employee is working on the company grounds, and the employee does not have to worry about any possible data leaks once he leaves the company grounds. Another scenario could utilize the *Time-Context*. This suits very well for an employee who is traveling a lot, and therefore, a location constraint does not make much sense. The individual can define a preference that allows data collection by the employer during certain time periods (see Listing 3). The example shows a ConTra preference, defined by an employee, which allows the collection of GPS coordinates and sensor data during working hours, i.e., from Monday until Friday between 7:00 am. and 4:00 pm., and the processing for any needed purpose by the employer. No other data are allowed to be collected, and only the employer himself has a processing right on the data, i.e., third party sharing is forbidden. As no personal information is claimed by this process, no transformation method is desired by the employee to mask his data, and therefore, the quality of collected data is maximized for the employer. While there are many more possible scenarios where ConTra could be utilized for the scope of this paper, we limit the focus to the examples given in this paragraph.

Listing 3 ConTra preference for an employee sharing GPS coordinates and sensor data during working hours from Monday to Friday for any needed processing purpose with the employer. The symbol * represents the placeholder *all*.

1 "preference" : {
2   "connective" : AND,
3   "rule" : {
4     "purpose" : {
5         "permitted" : ["E-Commerce"],
6         "excluded" : [*]
7     },
8     "data" : {
9         "permitted" : ["Address", "Financial Data"],
10         "excluded" : [ ]
11     },
12     "data" : {
13         "permitted" : ["GPS-Coordinates", "Sensor Data"],
14         "excluded" : [ ]
15     },
16     "context" : {
17         "startWeekday" : "Monday",
18         "endWeekday" : "Friday",
19         "startTime" : "07:00:00",
20         "endTime" : "16:00:00"
21     },
22     "transformation" : "no_method"
23   }
24 }

## 4. Privacy Interfaces

As most of the common privacy preference languages are built for a single specific privacy language, there is a limited applicability for each of them in real scenarios. On the one hand, there are several different privacy languages that can be used in real applications. On the other hand, not every privacy policy is built on a privacy language. These are only two possible reasons why preference languages are barely used. We hope by covering a bigger amount of applications with preferences, we can demonstrate its usefulness and therefore motivate for both a broader usage of privacy languages on the company side and preferences on the user side. To realize the requirement for privacy language compatibility, we introduce the principle of privacy interfaces in the following.

### 4.1. Concept

**Figure 3:**
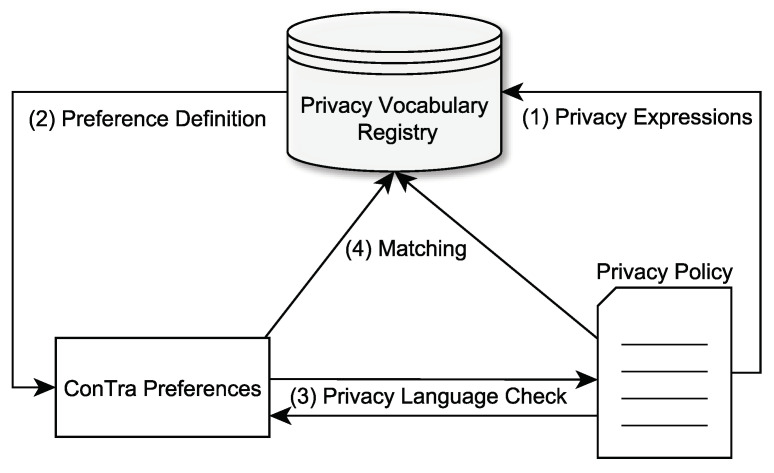
Workflow of the privacy interfaces process. Privacy language-specific terms and constructs are integrated in a shared registry and later used for matching between user preferences and privacy policy.

While there might be features that several privacy languages have in common, there are also plenty of unique concepts. This might include the underlying file format, elements, or vocabulary. In addition, each *Controller* might define expressions that are unique for a specific privacy policy. All these factors have to be considered when creating a preference language with broad privacy language compatibility. As it is almost impossible to create a single preference that can negotiate with any privacy language, we decided to add an additional layer to this process: the privacy interfaces. This way, measures can be taken to enable privacy language-independent preference negotiation (see Figure 3). The *Privacy Vocabulary Registry* is the core component of the privacy interfaces principle. It is a registry system located on a web server and based on a standardized privacy vocabulary. The latter will be discussed in the following section.

It must be mentioned that each privacy language, which should be supported by the privacy interfaces, first needs to be linked to the registry. This setup process is completed by experts and only has to be completed once for each privacy language. Afterwards, every privacy policy based on the integrated languages can be used for ConTra negotiations. Only unique expressions of a *Controllers’* privacy policy still need to be linked if the privacy policy is not based on a standardized vocabulary. Once a privacy language-based privacy policy is created by a *Controllers’* privacy protection officer, it can be linked to the *Privacy Vocabulary Registry*. Therefore, the registry is extended by entries that link the *Controllers’* unique privacy expressions to the equivalent expression of the privacy vocabulary. In the next step, the privacy vocabulary is used to create unified ConTra preferences. These preferences are purely based on the privacy vocabulary and are therefore independent of privacy language or privacy policy-specific expressions and are capable of negotiating with any policy that is linked to the *Privacy Vocabulary Registry*. Once a user enters a website, it is checked if the privacy language has been integrated in the *Privacy Vocabulary Registry* in advance and if the privacy policy has been linked in case of unique expressions. If both conditions are true, the preferences and the privacy policy are matched against each other by querying the registry. Otherwise, the user is informed that no matching is possible. The whole process requires the *Controllers* to do some additional work in order to create preference-ready privacy policies, but they gain added value by using standardized vocabularies fostering interoperability and accountability. Users only have to do one single preference setup and do not have to worry about different preferences languages or tools. So, the responsibility lies at the *Controllers* mostly, and users can focus on simplified policy negotiations supported by a broad amount of applications.

### 4.2. Vocabulary

With plenty of privacy languages being developed in the past, also a big diversity in integrated vocabularies exists. For example, P3P [4], as the first privacy language, came up with its own vocabulary. While the P3P vocabulary has been adopted by some other privacy languages such as PPL as part of the PrimeLife project [29], other projects such as SPECIAL [7] introduced their own vocabulary. Therefore, in order to make the privacy interface principle work and to enable privacy language compatibility, a unified privacy vocabulary is needed. At the time of writing this paper, no standardized vocabulary that defines expressions related to personal data handling, privacy policies and GDPR requirements exists. A first step into this direction was taken by the W3C Data Privacy Vocabulary and Controls Community Group (DPVCG) with the creation of the Data Privacy Vocabulary (DPV) [30]. Although it is not considered as a standard yet, it shows that the topic is of interest for companies and is a first big step into the right direction for a better future of privacy. It focuses on personal data handling and provides modular ontologies for data categories, purposes, processing categories, technical and organisational measures, legal basis, consent, and entities. Therefore, all important components for the definition of privacy policies and preferences are given while also considering the newly introduced GDPR requirements. Due to these reasons, the DPV is integrated in our prototypical implementation of the *Privacy Vocabulary Registry*.

## 5. Discussion

In this work, we analyzed the state of the art regarding the research question *“What are the requirements for a post-GDPR privacy preference language that enables the negotiation with privacy policies, and do state-of-the-art preference languages fulfill these requirements?”* With modern technologies emerging over the last couple of years, such as the rapid rise of mobile and smart technologies, and the enactment of the GDPR [12] in Europe, most of the existing preference languages are technically or legally outdated due to the changed requirements. These preference languages are usually designed to fulfill a specific purpose, such as CPL [21] introducing preferences for context information but omitting purpose-based processing or SecPAL4P [20] achieving high expressiveness but lacking usability and legal considerations. To the best of our knowledge, no preference language exists that unites all this functionality within one single framework and therefore meets all modern requirements. We showed that there is a research gap for preference languages in terms of privacy language compatibility in order to gain a broad coverage of applications with a single preference setup. Finally, none of the discussed preference languages offers preference-based consent recommendations based on the gradation for policy negotiation automation levels (see Section 2.1), leading to a second research gap.

ConTra is our approach to tackle these research gaps. It offers great opportunities to improve the user experience at managing privacy for both mobile scenarios and normal internet browsing. In general, *Controllers* can benefit from a properly conducted preference setup, as it allows for a faster and better informed policy negotiation. This means that instead of rejecting all optional purposes by pressing the according button, users might be willing to share specific information more often. Employees could utilize location preferences to limit information sharing while being on the company grounds or time preferences to limit information sharing to working hours if they need to be mobile. In addition, the generic and modular nature of ConTra preferences guarantees both expressiveness and extensibility while the definition of preferences in JSON format maintains usability by human-readable rules and allows for efficient processing. Therefore, ConTra can also be applied in resource-limited scenarios, e.g., IoT with edge devices, and can be adapted to other domains. The consent recommendation feature of ConTra allows for fast policy negotiation, whereas preferences can be altered or new preferences can be created during the process in order to handle future negotiations even faster. Finally, ConTra’s unique privacy language compatibility is enabled by interposing privacy interfaces between preference and policy negotiation to achieve a broader applicability for privacy preferences. Therefore, ConTra meets all requirements of a modern preference language, resulting in the most complete preference framework yet to our knowledge.

Nevertheless, there are some limitations of the approach. In order to enable unified preference matching with ConTra, the initial integration of a privacy language in the *Privacy Vocabulary Registry* is required. Even though this integration only has to be conducted once per privacy language, it could be a critical step in real applications, whereas stakeholders pass on the work to each other. While the concept of ConTra enables a broader applicability of preferences among privacy languages, it is also limited to this scenario at the current state. Therefore, privacy policies, which are not based on a privacy language, cannot be negotiated with ConTra preferences, e.g., plain-text policies. As the integrated DPV privacy vocabulary is not considered as a standard yet, the semantics of ConTra and the *Privacy Vocabulary Registry* might be changed in the future if a (possibly global) standard for privacy expressions emerges.

Finally, we hope to motivate further research in the field of privacy preference handling by our work. We showed that there is a huge gap when it comes to a unified application of privacy preferences. In addition, there is a lack of support of mobile technologies, while these technologies are dominating the current market. In general, there is almost no real application of privacy preferences: either users are not willing to invest time for a detailed preference setup, or there is a lack of support from *Controllers*. It is essential that users learn about the importance of privacy and measures they can take to protect their personal data as well as companies being willing to adapt privacy-preserving technologies.

## 6. Conclusions

Although several preference languages have been developed over the last two decades, each of them is specifically designed to complement exactly one privacy language. As many diverse privacy languages exist, we found that there is a huge gap in terms of privacy language compatibility by existing preference languages. To the best of our knowledge, none of the existing preference languages support this criterion. This is a problem when it comes to the application of preferences, as users would expect that their preferences work on most of the websites, which is not the case in current implementations. Therefore, in this paper, we proposed ConTra, a preference language for **Con**sent and **Tra**nsparency, as a novel approach to privacy preference handling. It intends to close the gap between user preferences and privacy policies by providing privacy language compatibility at policy negotiation.

To tackle this challenge, we introduce the principle of privacy interfaces to enable unified preferences based on a privacy vocabulary. Unique privacy expressions of *Controllers* will be integrated in a *Privacy Vocabulary Registry* and will be assigned to the corresponding entry of the privacy vocabulary. As a result, ConTra preferences can be matched against any expressions specific to a privacy language if these expressions have been registered in the *Privacy Vocabulary Registry*. This way, parts of the workflow are shifted from the users to the experts. In addition to privacy language compatibility, ConTra features contain all the functionalities required by a modern preference language. This includes the support of context information, such as location and time constraints for mobile devices, expressiveness, extensibility, and legal requirements, such as the explicit consent and transparency of policy actions as claimed by the European GDPR, while also achieving human-readable preference rules. Although ConTra is specifically designed to meet the European legal situation, it also should be applicable for other regions if the processing of data is based on purposes. However, it remains an open question if a complete adaptation, e.g., for American or Asian privacy principles, is possible.

For the future, we envision to assemble the ConTra framework step by step. A validation algorithm will ensure that ConTra preference rules do not cause any contradictions if joined together by connectives. In addition, the privacy interfaces will be implemented and evaluated based on some prominent privacy languages by creating proper use cases. Furthermore, the framework will be extended by consent recommendation at policy negotiation to close the corresponding research gap. A user-driven evaluation will refine the *ConTra Negotiation UI* and the *ConTra Consent Recommender UI*. We also desire to implement a machine-learning approach to analyze plain-text privacy policies and create representations, which can be negotiated with ConTra preferences, in order to cover most occurrences of privacy policies.

## Figures and Tables

**Table 1:** Overview of requirements for a novel preference language and which languages meet them. The requirements without any particular ordering are *privacy language compatibility (pl comp)*, *GDPR compliance*, *transparency*, *human-readability* (readable), *expressiveness*, *context information* and *extensibility*.

	Pl Comp	GDPR	Transparency	Readable	Expressiveness	Context	Extensibility
APPEL			✓				
XPref			✓				
SemPref			✓	✓			
SecPAL4P			✓		✓		✓
PPL		✓	✓		✓	✓	✓
CPL						✓	✓
YaPPL		✓		✓	✓		✓

## Data Availability

Not applicable.
